# Supplementary material for: Extracorporeal Shockwave Therapy (ESWT) Alleviates Pain, Enhances Erectile Function and Improves Quality of Life in Patients with Chronic Prostatitis/Chronic Pelvic Pain Syndrome
Source: J Clin Med. 2021 Aug 16;10(16):3602. doi: 10.3390/jcm10163602 (PMC8396816; doi:10.3390/jcm10163602)
Supplement: Supplementary file 1 [file jcm-10-03602-s001.zip › jcm-1320718-supplementary.pdf]

Supplementary Table S1. Baseline and time-phased changes in NIH-CPSI, IIEF-5, EHS, IPSS and AUA QOL_US Scores in participants (*n* = 215).

Parameters	Baseline	pESWT_1	pESWT_2	pESWT_6	pESWT_12
CPSI pain domain	12.53±3.25	8.64±4.27	7.72±4.19	7.5±3.71	5.41±3.57
CPSI urinary domain	4.98±2.72	3.12±2.15	2.8±2.00	2.96±1.86	2.26±1.99
CPSI QoL domain	9.62±2.06	6.86±2.83	6.47±2.93	6.96±2.69	4.93±2.61
CPSI total score	27.10±6.81	18.62±8.22	17±8.01	17.42±7.13	12.57±7.30
IIEF-5	15.82±7.70	18.43±6.34	20.42±5.59	20.25±5.94	18.65±6.85
EHS	3.11±0.99	3.37±0.65	3.42±0.58	3.75±0.45	3.32±0.85
IPSS	13.9±8.41	10.14±8.32	8.62±5.80	8.06±4.51	6.83±5.14
QoL	4.29±1.54	3.26±1.93	3.45±2.34	3.25±1.69	2.6±1.56

CPSI/NIH-CPSI = National Institutes of Health Chronic Prostatitis Symptom Index; SD = standard deviation; QoL = quality of life; VAS = visual analog scale; IPSS = International Prostate Symptom Score; QoL/AUA QOL_US = American Urological Association Quality of Life Due to Urinary Symptoms; IIEF = International Index of Erectile Function; EHS = erection hardness score; ESWT = extracorporeal shockwave therapy; pESWT = post extracorporeal shockwave therapy
